# Evaluation and comparison of Loop-mediated Isothermal Amplification (LAMP), PCR and nested PCR for detection of Ehrlichia canis in naturally infected dogs

**DOI:** 10.1186/1753-6561-8-S4-P140

**Published:** 2014-10-01

**Authors:** Vitor Pinhanelli, Péricles Costa, Ana Lúcia Fachin, Mozart Marins

**Affiliations:** 1 Unidade de Biotecnologia, Universidade de Ribeirão Preto (UNAERP), Brazil

## Background

The detection of parasites in blood samples by DNA based assays is mostly performed by using the PCR technique, but due to the time of the reactions and costs involved, they still cannot be used in large-scale in routine laboratories. Recently, an alternative are assays based on the technique of Loop-mediated Isothermal Amplification (LAMP), which requires a shorter reaction time, higher specificity and has a lower cost [1]. This study aimed to develop a LAMP assay for the detection of *Ehrlichia canis*, a Gram-negative endobacteria and the etiologic agent of canine monocytic ehrlichiosis (CME), a major disease transmitted by ticks to dogs [2]. DNA extracted from blood samples of dogs presented to veterinary clinics and laboratories in the city of Ribeirão Preto, showing clinical signs indicative of EMC, were used in the assays. The samples were also previously diagnosed by PCR for presence of E.ca*nis*. Two sets of LAMP primers were used for the amplification of a fragment of the genes p30 (P30) and Heat-shock operon groESL (GRO).

## Methods

The LAMP reactions were performed according to published protocols [3]. Briefly, each LAMP reaction mixture contained 1 µl extracted DNA, 20 pmol of each FIP and BIP primer, 5 pmol of each F3 and B3 primer, 0.5 mM of each dNTP, and 1X ThermoPol reaction buffer (20 mM Tris-HCl, 10 mM KCl, 10 mM (NH4)2SO4, 2 mM MgSO4, 0.1% Triton X-100, pH 8.8). The final volume was adjusted to 19 µl with autoclaved ultrapure water and the mixture was incubated at 95°C for 2 min, followed by incubation at ice. After the addition of 8 units Bst DNA polymerase large fragment (New England Biolabs, UK), the reactions were incubated at 60°C for 60 min and subsequently at 80°C for 10 min to terminate the reaction. A 10-µl aliquot of each reaction was used for electrophoresis on 2.5% agarose gel in Tris-acetic acid-EDTA (TAE) buffer and visualized under UV light after staining with ethidium bromide.

## Results and conclusion

In this preliminary tests of a LAMP assay for detection of *E. canis *in canine blood samples, the primers directed to the p30 gene presented a better performance than the ones directed to the Heat-shock operon groESL (GRO). The p30 primer set was able to amplify the target gene from samples obtained from different regions of Brazil.

## Aknowledgements

This study was supported by grants from Fundação de Amparo a Pesquisa do Estado de São Paulo (2012/02920-7 and 2009/12419-0) and CAPES.
